# Supplementary material for: Accurate prediction of the optical properties of nanoalloys with both plasmonic and magnetic elements
Source: Nat Commun. 2024 Jan 27;15:834. doi: 10.1038/s41467-024-45137-x (PMC10821890; doi:10.1038/s41467-024-45137-x)
Supplement: Supplementary file 1 — Supplementary Information [file 41467_2024_45137_MOESM1_ESM.pdf]

Supplementary information

Accurate prediction of the optical properties of nanoalloys with both plasmonic and magnetic elements

Vito Coviello,[1] Denis Badocco,[1] Paolo Pastore,[1] Martina Fracchia,[2,3] Paolo Ghigna,[2,3] Alessandro Martucci,[3,4] Daniel Forrer,[1,5,]* Vincenzo Amendola[1,3]*

[1] Department of Chemical Sciences, Università di Padova, via Marzolo 1, 35131 Padova, Italy

[2] University of Pavia, Department of Chemistry, viale Taramelli 16, 27100 Pavia, Italy

[3] INSTM, National Inter-University Consortium for Materials Science and Technology, Via G. Giusti 9, 50121 Florence, Italy

[4] Department of Industrial Engineering, University of Padova, Via Marzolo 9, 35131 Padova, Italy

[5] CNR – ICMATE, via Marzolo 1, 35131 Padova, Italy

* e-mail: daniel.forrer@unipd.it; vincenzo.amendola@unipd.it

Contents

Suppl. Note 1 **Additional STEM-EDX analysis on Au-Co nanoalloy**

Suppl. Note 2 **Parameterization of the Hubbard term and comparison with the experimental dielectric functions of pure metals**

Suppl. Note 3 **EXAFS analysis on Au-Co nanoalloys**

Suppl. Note 4 **Comparison of DFT+U dielectric functions using SRO- and SRO+ models**

Suppl. Note 5 **Comparison of the mass extinction coefficients of Au-Co nanoalloys calculated with the LDA+U and PBE functionals**

Suppl. Note 6 **Comparison of calculated and experimental dielectric functions of bulk alloys**

Suppl. Note 7 **PDOS of Au-Co alloy models**

Suppl. Note 8 **Additional characterization data of NPs samples**

Suppl. Note 1
Additional STEM-EDX analysis on Au-Co nanoalloys

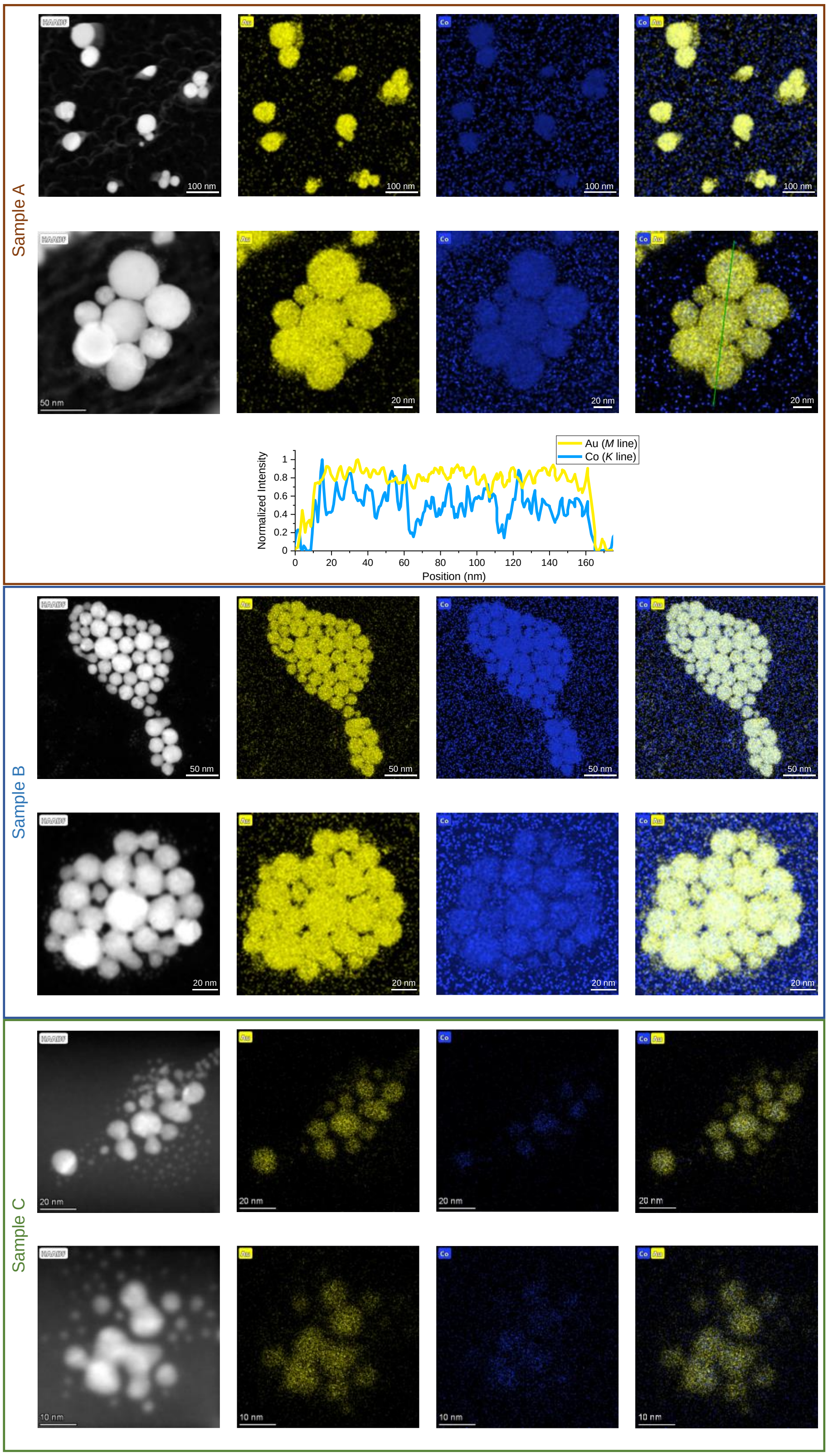

Supplementary Figure 1. Additional STEM-EDX maps on Au-Co NPs. Au M line and Co K line maps showing that the NPs do not have a core-shell structure and are homogeneous alloys. The linescan profile of Au and Co in a group of nanoparticles of sample A is also shown. Au: yellow; Co: blue.

Sample A					Sample B					Sample C				
**NP**	**Au (at%)**	**EDX error**	**Co (at%)**	**EDX error**	**NP**	**Au (at%)**	**EDX error**	**Co (at%)**	**EDX error**	**NP**	**Au (at%)**	**EDX error**	**Co (at%)**	**EDX error**
1	74	13	26	5	1	79	15	21	3	1	86	11	14	2
2	79	14	21	4	2	79	15	21	3	2	85	10	15	2
3	75	13	25	5	3	78	14	22	3	3	83	10	17	3
4	78	14	22	4	4	77	14	23	4	4	83	10	17	3
5	76	14	24	5	5	81	15	20	3	5	91	11	9	2
6	73	13	27	5	6	76	14	24	4	6	93	11	8	3
7	73	13	27	5	7	76	14	24	4	7	85	10	15	3
8	74	14	26	4	8	79	15	21	3	8	92	11	8	2
9	76	14	24	4	9	84	16	17	3	9	80	10	19	3
10	78	15	22	4	10	78	14	22	4	10	90	12	12	2
11	74	14	26	4	11	81	15	19	3	11	88	12	13	2
12	77	15	23	4	12	78	14	22	3	12	81	10	18	3
13	76	14	24	4	13	76	14	24	4	13	82	10	17	3
14	74	14	26	4	14	75	12	25	5	14	82	10	18	3
15	75	14	25	4	15	77	13	23	4	15	86	11	15	3
16	72	13	28	4	16	77	13	23	4	16	80	10	19	3
17	76	14	24	4	17	78	13	23	4					
					18	76	13	24	4					
					19	78	13	22	4					
					20	76	13	24	4					
					21	77	13	23	4					
					22	76	13	24	5					
					23	78	13	23	4					
**Mean**	**75**	**± 14**	**25**	**± 4**	**Mean**	**78**	**± 14**	**22**	**± 4**	**Mean**	**85**	**± 11**	**15**	**± 3**

Supplementary Table 1. STEM-EDX quantitative analysis on single Au-Co NPs. Values obtained from Au L line and Co K line.

Suppl. Note 2
Parameterization of the Hubbard term and comparison with the experimental dielectric functions of pure metals

LDA+U parameterization was performed by computing the dielectric function of elemental Au, Co, Fe, Ag and Cu and comparing it with experimental curves from literature. Experimental dielectric functions of Au was taken from Ref.[1], those of Ag and Cu from Ref. [2], those of Co and Fe from Ref. [3].
All structures were optimized at the PBE level through variable-cell relaxations. In Supplementary Figure 2 the behavior of several exchange-correlation functionals is shown for the Au case. The LDA+U functional with U = 2.0 eV provides the best result.
PBE and SCAN results are close to pure LDA calculations but perform worse than LDA+U with U = 2.
GLLBSC and TB09 performance are close to LDA+U but suffer of numerical instability that prevent their use in magnetic materials and where not considered for this study.
The optimal U parameters for the LDA+U calculations were identified by comparing computed and experimental dielectric functions. Plots are shown in Supplementary Figure 3 and R^2 values between computed and calculated dielectric functions are reported in Supplementary Table 2 for selected values of U. LDA+U results are systematically better than PBE ones. In the Ag case the Hubbard correction was applied to both 4*d* and 6*p* orbitals to improve the match with the experiment.
The dielectric functions of elemental Au, Co, Fe, Ag and Cu computed with the LDA+U are compared also with experimental curves from ellipsometry measurements performed in this work (Supplementary Figure 4).

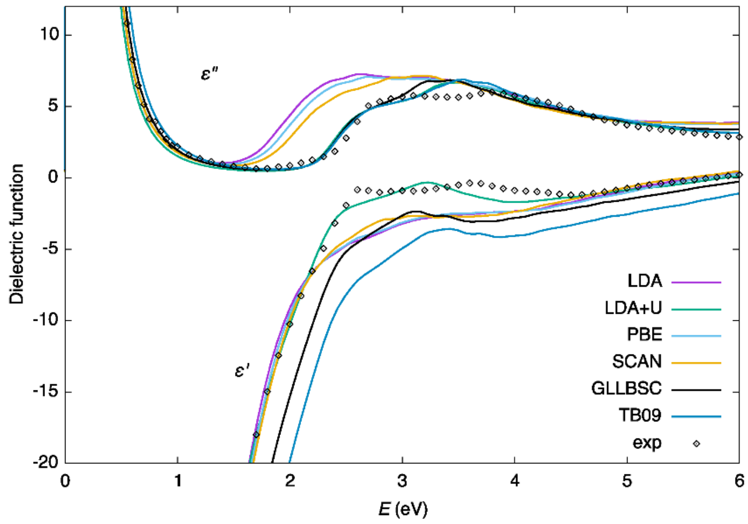

Supplementary Figure 2. Dielectric function of Au computed with different functionals. The dielectric function of Au is compared to the experimental value from Ref.[1]. Colors in the graphs are: black hollow dots (experimental), blue (TB09), black (GLLBSC), orange (SCAN), light blue (PBE), green (LDA+U), purple (LDA). Source data are provided as a Source Data file.

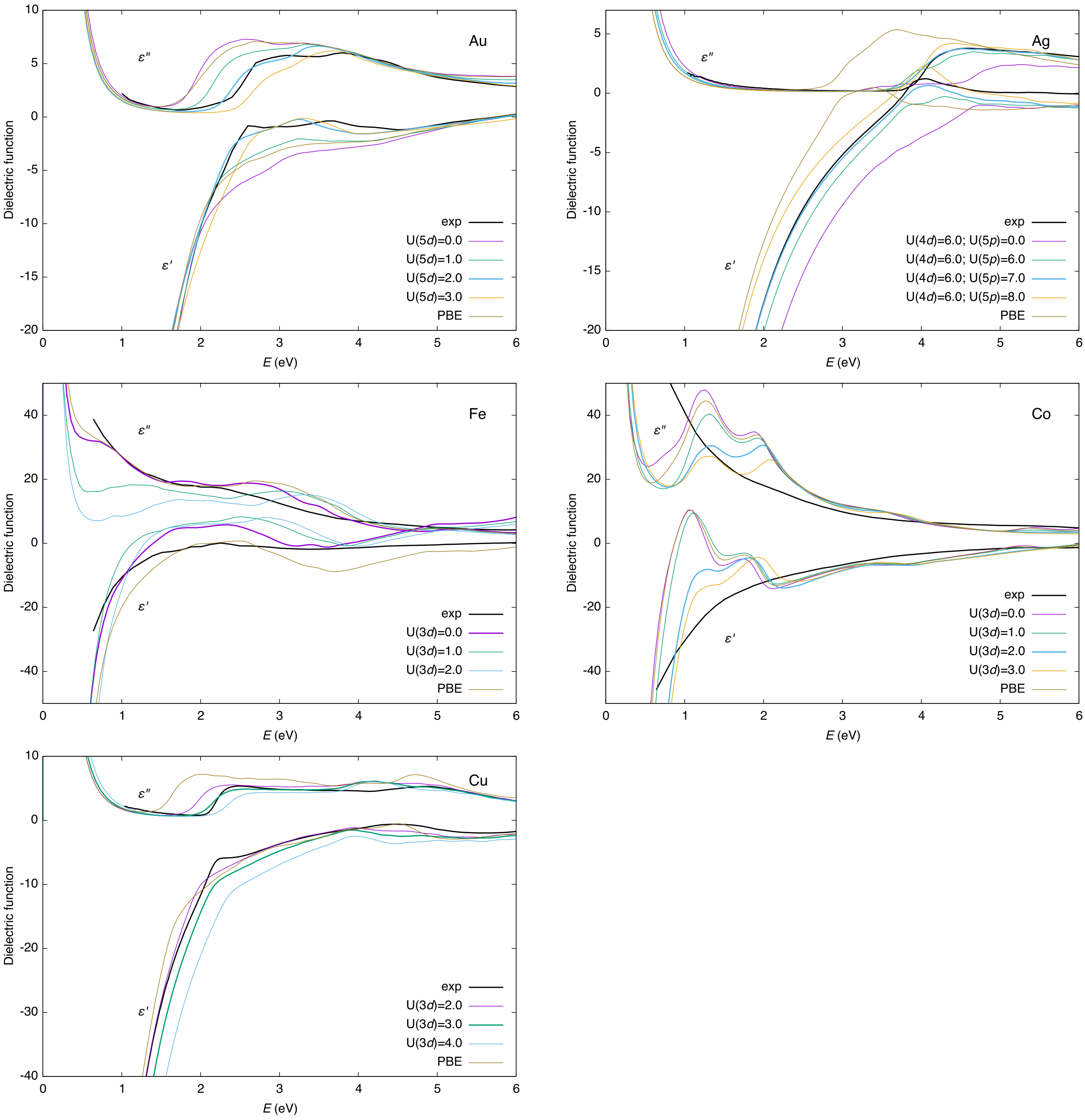

Supplementary Figure 3. Calculated dielectric functions for selected values of the U parameter. PBE results are also shown. Au: black (experimental), purple (U(5*d*) 0.0), green (U(5*d*) 1.0), light blue (U(5*d*) 2.0), orange (U(5*d*) 3.0), brown (PBE). Ag: black (experimental), purple (U(4*d*) = 6.0; U(5*p*) = 0.0), green (U(4*d*) = 6.0; U(5*p*) = 6.0), light blue (U(4*d*) = 6.0; U(5*p*) = 7.0), orange (U(4*d*) = 6.0; U(5*p*) = 8.0), brown (PBE). Fe: black (experimental), purple (U(3*d*) 0.0), green (U(3*d*) 1.0), light blue (U(3*d*) 2.0), brown (PBE). Co: black (experimental), purple (U(3*d*) 0.0), green (U(3*d*) 1.0), light blue (U(3*d*) 2.0), orange (U(3*d*) 3.0), brown (PBE). Cu: black (experimental), purple (U(3*d*) 2.0), green (U(3*d*) 3.0), light blue (U(3*d*) 4.0), brown (PBE). Source data are provided as a Source Data file.

		LDA+U				PBE
**Au**	U value	0 eV	1 eV	2 eV	3 eV	-
	R^2 ε'	0.9414	0.9768	0.9993	0.9887	0.9798
	R^2 ε"	0.3346	0.7518	0.9624	0.8495	0.4508
	R^2 average	0.6380	0.8643	0.9809	0.9191	0.7153
**Co**	U value	0 eV	1 eV	2 eV	3 eV	-
	R^2 ε'	-1.4981	-0.4605	0.6582	0.6840	-1.2694
	R^2 ε"	0.5439	0.1389	-0.0915	-0.2506	0.3776
	R^2 average	-0.4771	-0.1608	0.2834	0.2167	-0.4459
**Fe**	U value	0 eV	1 eV	2 eV		-
	R^2 ε'	0.6661	0.5603	0.5820		0.6473
	R^2 ε"	0.9412	0.2449	-1.2357		0.9055
	R^2 average	0.8037	0.4026	-0.3269		0.7764
**Ag**	U value	*d* 0 eV; *p* 0 eV	*d* 6 eV; *p* 6 eV	*d* 6 eV; *p* 7 eV	*d* 6 eV; *p* 8 eV	-
	R^2 ε'	0.7897	0.9781	0.9993	0.9395	0.8930
	R^2 ε"	0.0669	0.9637	0.9783	0.9462	0.1322
	R^2 average	0.4283	0.9709	0.9888	0.9428	0.5126
**Cu**	U value	0 eV	2 eV	3 eV	4 eV	-
	R^2 ε'	0.9665	0.9983	0.9496	0.7718	0.9804
	R^2 ε"	0.1747	0.7621	0.9322	0.7891	-0.0391
	R^2 average	0.5706	0.8802	0.9409	0.7804	0.4707

Supplementary Table 2. R^2 values of the comparison between calculated and experimental dielectric functions. R^2 for teal and imaginary components and the average of the two results are reported for selected values of the Hubbard parameter U. The best matches are highlighted in green. U = 0 corresponds to pure LDA results. PBE results are also reported.

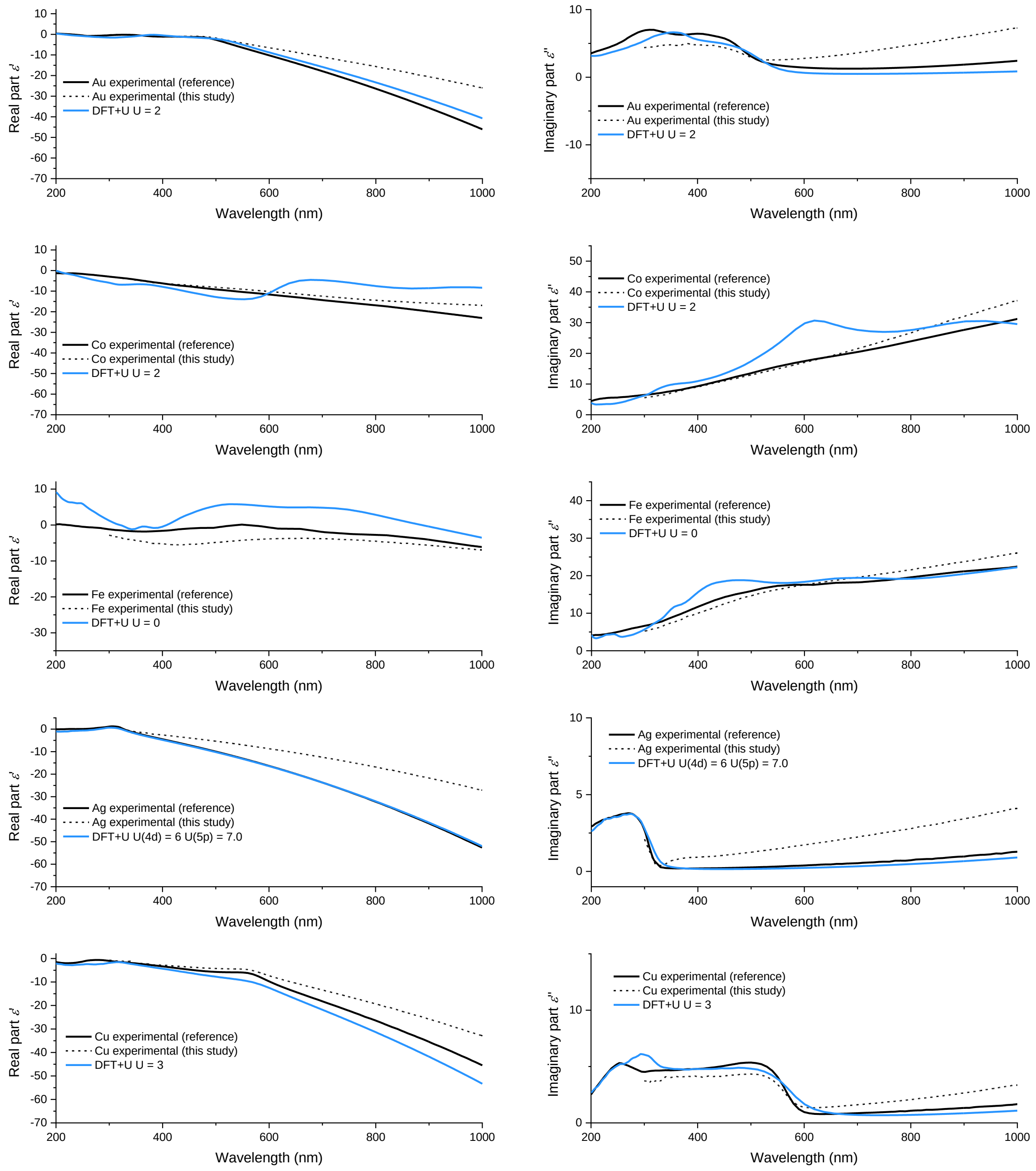

Supplementary Figure 4. Comparison of computed and experimental dielectric functions. Comparison of the dielectric functions of elemental Au, Co, Fe, Ag and Cu computed with the LDA+U (blue) with experimental curves from Ref. [1] for Au, [3] for Co and Fe, [2] for Ag and Cu (black continuous line) and from ellipsometry measurements performed in this work (black dashed lines). Source data are provided as a Source Data file.

Suppl. Note 3
EXAFS analysis on Au-Co nanoalloys

The XANES (X-ray absorption near-edge structure) spectra of samples A, B and C at the Au-L_3 edge are displayed in Supplementary Figure 5 and compared to the spectrum of metallic Au foil, employed as reference. All the spectra show a close resemblance to the reference spectrum, as expected for alloys that retain the FCC structure. The main features appear smeared out, which is indicative of the presence of nanoparticles. The minor differences between the spectra of the three samples can be attributed to the different sizes of the nanoparticles in the three cases.

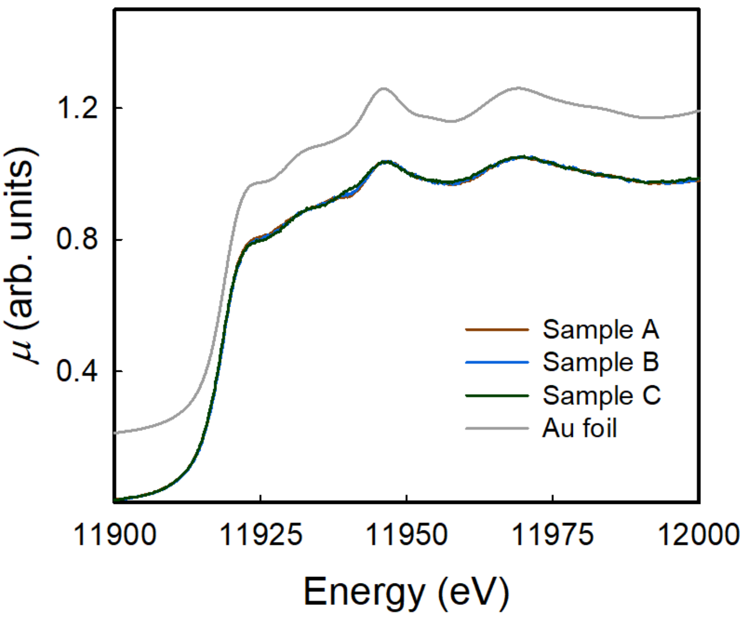

Supplementary Figure 5. XANES spectra. The XANES spectra of Au L_3-edge of samples A (brown), B (blue) and C (green), compared to the spectrum of Au foil (grey), employed as reference. The latter is stacked on the y-axis for better comparison. Source data are provided as a Source Data file.

To gain insights into the local structure of the samples, the EXAFS (extended X-ray absorption fine structure) signals were extracted, and the corresponding Fourier Transform (FT) calculated (see Supplementary Figure 6). From the FTs, it can be observed that the signal rapidly drops after ca. 3 Å, indicating that, as expected from nanoparticles, only the first neighbouring shell contributes to the EXAFS. All signals were therefore refined using a structural model, with a central Au atom as photoabsorber and one accounting for the Au-*M* distance, where *M* is the atom in the surrounding shell. One Au-*M* shell was enough for the fit, thus avoiding unnecessary parameters due to a second shell. Since alloying with Co is expected to cause a contraction of the lattice parameter and, consequently, of the first-shell distance, the amount of Co bonded with Au is related to the value of the refined distance. The structural parameters after refinement are shown in Supplementary Table 3 and the first-shell distances obtained after refinement are plotted in Supplementary Figure 7 as a function of the experimentally measured Co atomic percentage of the three samples. In all cases, the first-shell distances are lower than the crystallographic one expected for Au FCC, confirming alloying with Co. Among the three, sample C shows the largest distance, indicating a lower amount of Co alloyed with Au. Samples A and B have the same first-shell distances within the experimental error, suggesting that a comparable amount of Co is bonded to Au, despite the higher nominal Co content in sample A. This is in agreement with short-range Co segregation in the NPs of sample A.

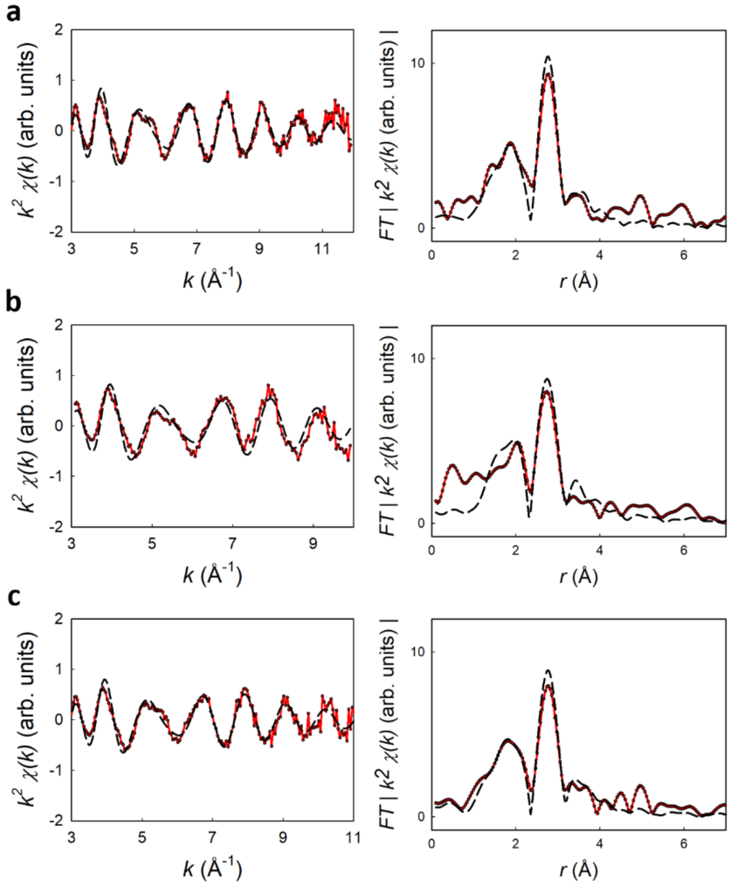

Supplementary Figure 6. EXAFS analysis. EXAFS (left panels) signal and corresponding Fourier Transform (right panels) for samples A (**a**), B (**b**) and C (**c**). The red dotted lines represent the experimental data, while the black dashed line is the simulated curve. Source data are provided as a Source Data file.

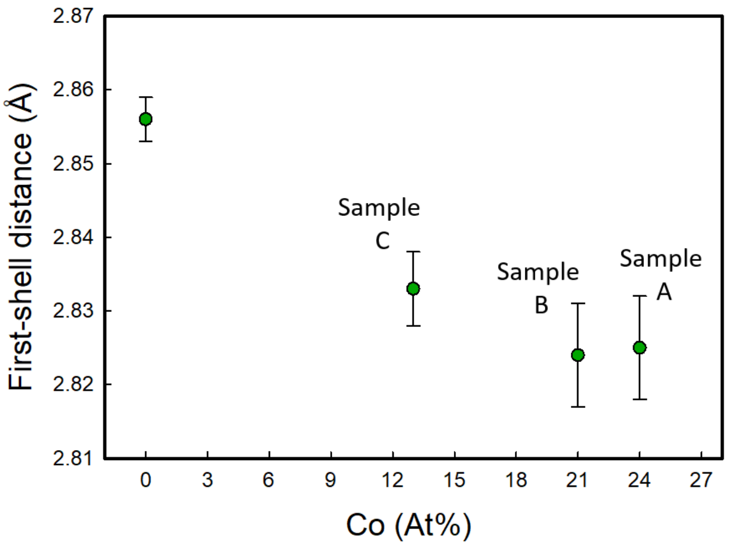

Supplementary Figure 7. First-shell distance. First-shell distance as a function of the Co content in the three samples. Source data are provided as a Source Data file.

**Sample**	**Shell**	**Coordination number (*N*)**	***R* (Å)**	***σ*^{2} (Å^2)**
A	1	12	2.825(7)	0.0114(6)
B	1	12	2.824(7)	0.012(1)
C	1	12	2.833(5)	0.0127(7)

Supplementary Table 3. EXAFS parameters. EXAFS parameters obtained from the fitting procedure. The error bars represent the fitting error.

Suppl. Note 4
Comparison of DFT+U dielectric functions using SRO- and SRO+ models

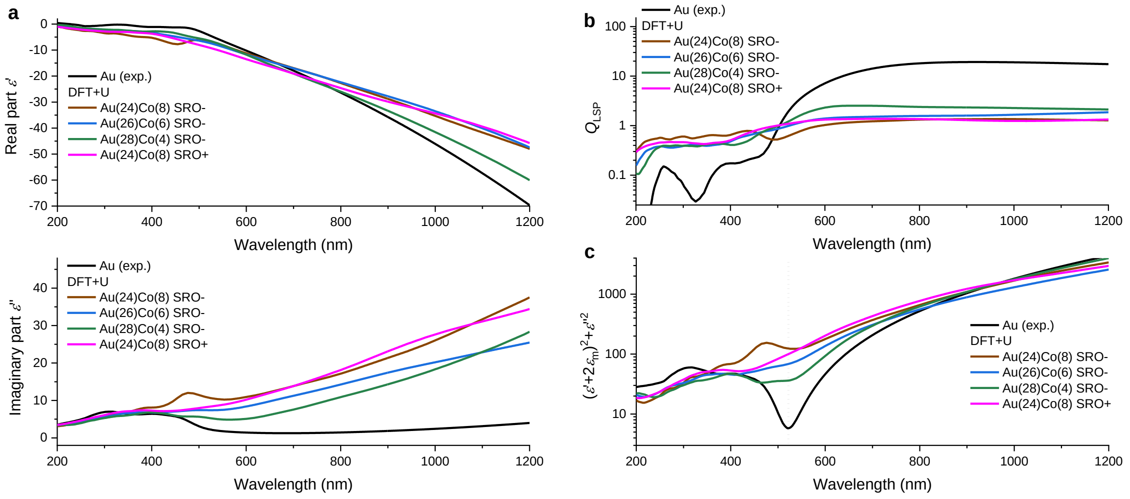

Supplementary Figure 8. DFT+U calculations for SRO- and SRO+ models. a ε' and ε'' obtained by DFT+U calculations with SRO- or SRO+ models, compared to the experimental value of pure Au. **b**-**c** Plots of Q_{LSP} (**c**) and $(\varepsilon'+2\varepsilon_{\mathrm{m}})^2 + \varepsilon''^2$ (complete Fröhlic condition in a nanosphere, **c**) using the calculated dielectric functions for the Au-Co alloy and the experimental value for Au. Colors in the graphs are: black (Au, experimental), brown (Au(24)Co(8) SRO-), blue (Au(26)Co(26) SRO-), green (Au(28)Co(4) SRO-), magenta (Au(24)Co(8) SRO+). Source data are provided as a Source Data file.

Suppl. Note 5
Comparison of the mass extinction coefficients of Au-Co nanoalloys calculated with the LDA+U and PBE functionals

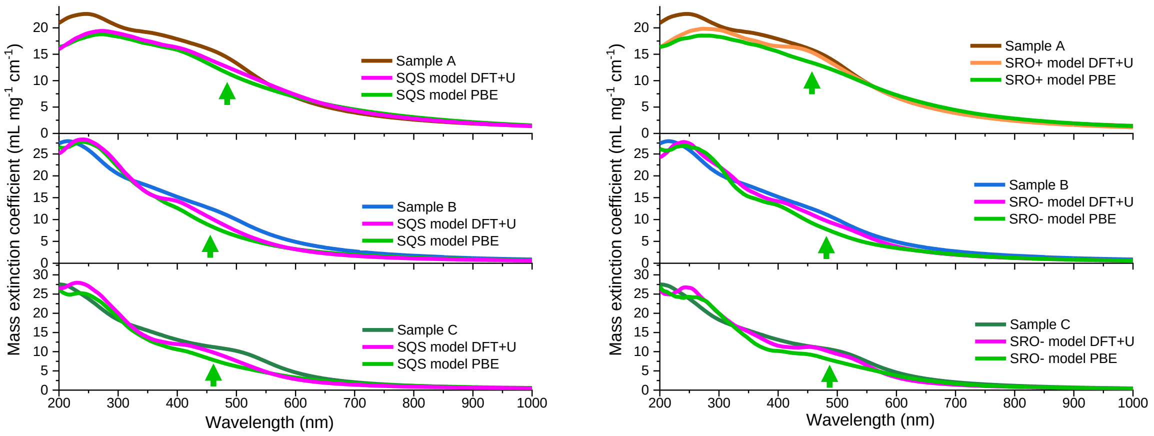

Supplementary Figure 9. Mass extinction coefficients calculated with the LDA+U and PBE functionals. Comparison of mass extinction coefficients calculated with the LDA+U and PBE functionals for the Au-Co samples (left: SQS cells; right: SRO- and SRO+ cells). Colors in the graphs are: brown (sample A), blue (sample B), green (sample C), magenta (LDA+U calculations for SRO- cells), orange (LDA+U calculations for SRO+ cell), light green (PBE calculations). The arrows (light green) indicate the region of the plasmon resonance in the nanoalloys, where the PBE calculations resulted in lower accuracy than the LDA+U functional, as expected from Ref.[4] and in agreement with the results obtained with pure Au (see Supplementary Figure 2). Note that the numerical stability of more recent functionals such as the GLLBSC one does not support a plasmonic alloy with magnetic elements.[5–8] In fact, our efforts to calculate the ground state of a 2x1x1 FCC supercell with composition Au_7Co_1 using the GLLBSC was unsuccessful, irrespectively of the chosen minimization algorithm and density mixing scheme. For this reason, the GLLBSC functional was not considered in this study. Source data are provided as a Source Data file.

Suppl. Note 6
Comparison of calculated and experimental dielectric functions of bulk alloys

The dielectric functions of two magnetic-plasmonic bulk alloys (Au(70)-Co(30) and Au(73)-Fe(27)) and two plasmonic bulk alloys (Au(40)-Ag(60) and Au(75)-Cu(25)) were calculated with the DFT+U model and compared to the experimental values reported in literature[9,10] or obtained in this study (Supplementary Figure 10). The results of PBE calculations with the same cell models are also shown. The dielectric functions calculated with the DFT in Ref.[6] for the Au(40)-Ag(60) and Au(75)-Cu(25) alloys are also reported.
The R^2 are reported in Supplementary Table 4. Note that the result for the Au-Co alloy (R^2 of 0.980 and 0.886 for ε' and ε'', respectively) provides further confirmation that the choice of U = 2 for Co is appropriate. Hence, although the optimization of U on the dielectric function of a bulk alloy with a composition similar to the NPs is easier, the use of the pure element dielectric functions also leads to appropriate results.

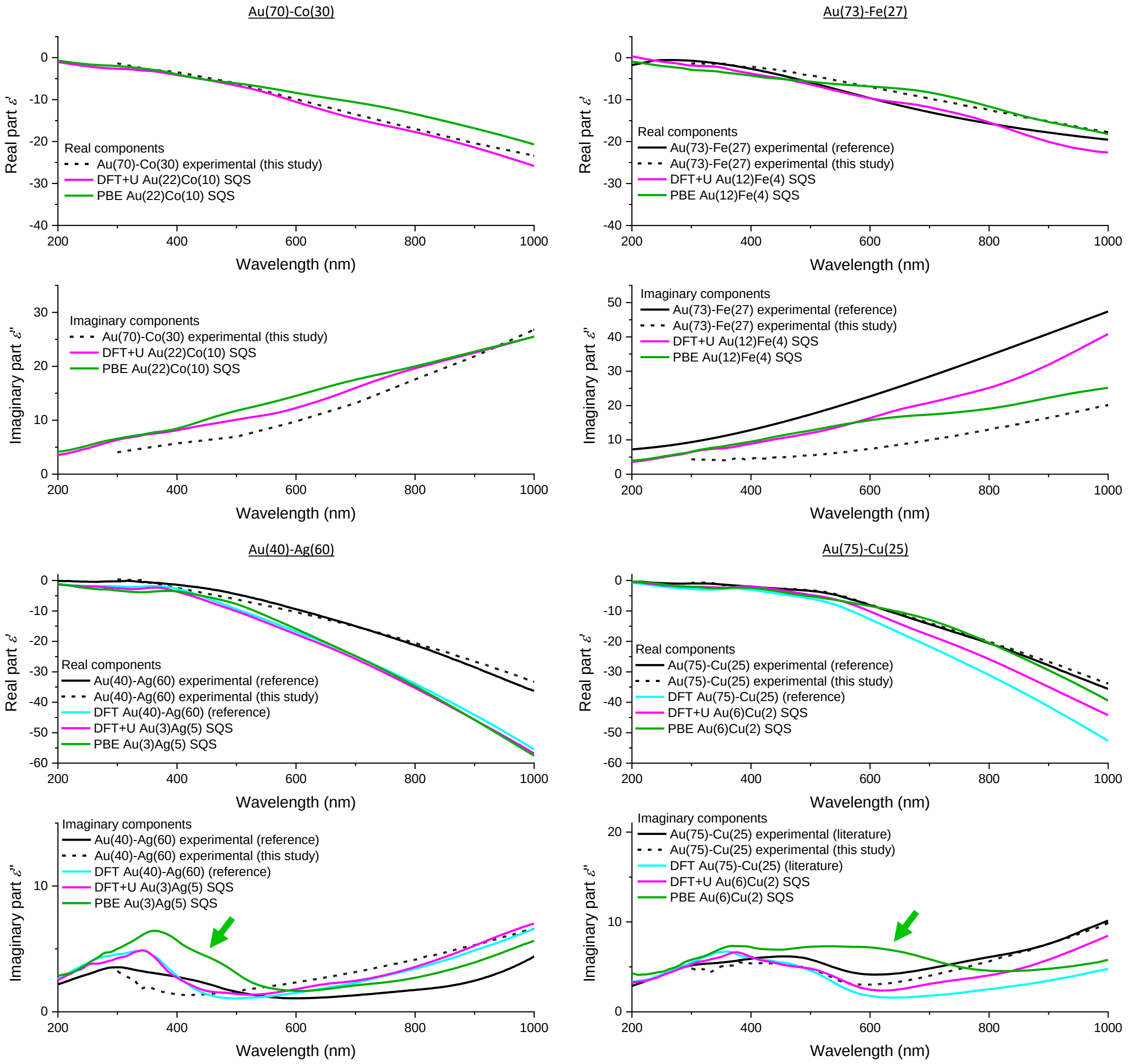

Supplementary Figure 10. Calculated and experimental dielectric functions of bulk alloys. Comparison of the experimental dielectric functions of two magnetic plasmonic bulk alloys (Au(70)-Co(30) and Au(73)-Fe(27)) and two plasmonic bulk alloys (Au(40)-Ag(60) and Au(75)-Cu(25)) with those calculated with the DFT+U model, the PBE model and the DFT model from Ref.[6]. The green arrows indicate the failure of the PBE model to reproduce the edge of the interband transitions in Au(40)-Ag(60) and Au(75)-Cu(25) alloys. Colors in the graphs are: black continuous line (experimental dielectric constants from Ref. [9] for Au(73)-Fe(27), [10] for Au(40)Ag(60) and Au(75)Cu(25)), black dashed line (experimental from this study), magenta continuous line (calculated with

DFT+U), green continuous line (calculated with PBE), cyan line (calculated from Ref. [6]). Source data are provided as a Source Data file.

**Au(70)-Co(30)**	***R*² – *ε*'**	***R*² – *ε*"**	**Average *R*²**
DFT+U	0.979972	0.885638	0.932805
PBE	0.874337	0.749372	0.811854
**Au(73)-Fe(27)**	***R*² – *ε*'**	***R*² – *ε*"**	**Average *R*²**
Experimental (this study)	0.843821	0.154102	0.498961
DFT+U	0.959394	0.897897	0.928645
PBE	0.822743	0.822908	0.822826
**Au(40)-Ag(60)**	***R*² – *ε*'**	***R*² – *ε*"**	**Average *R*²**
Experimental (this study)	0.983713	-3.63261	-1.32445
DFT (reference)	0.257914	-1.51579	-0.62894
DFT+U	0.095838	-2.11046	-1.00731
PBE	0.157174	-2.19334	-1.01808
**Au(75)-Cu(25)**	***R*² – *ε*'**	***R*² – *ε*"**	**Average *R*²**
Experimental (this study)	0.995869	0.728103	0.861986
DFT (reference)	0.468443	-2.30867	-0.92011
DFT+U	0.862169	0.125657	0.493913
PBE	0.984586	-0.850546	0.067020

Supplementary Table 4. R^2 values of the calculated and experimental dielectric functions. The R^2 for the calculated and experimental dielectric function reported in Supplementary Figure 10. Dielectric functions from DFT in Au(40)-Ag(60) and Au(75)-Cu(25) are from Ref. [6].

Suppl. Note 7
PDOS of Au-Co alloy models

Density of States (DOS) projected onto atomic orbitals (PDOS) derived from LDA+U calculations on SQS and SRO models of different alloy compositions are displayed in Supplementary Figure 11. Au 5*d* bands overlap significantly with Co 3*d* bands, thus a small hybridization between d orbitals of neighboring atoms is expected. Compared to SQS models, SRO- ones present sharper features in the *d*-band of both Au and Co. This is not surprising, because in SRO- structures all Co and Au atoms share a similar local environment, while in SQSs they are surrounded by different atomic arrangements. As a consequence, electron energy levels are less dispersed in the SRO- case. This is apparent also by looking at the imaginary part of the dielectric function (Supplementary Figure 12), where SRO- models show sharper features than the SQS models, due to a better alignment of energy states in the first case. Interestingly enough, PDOS of Au(24)Co(8) show spin-polarization in the *d*-bands of Au, which is not as apparent in the other cases. Also, the SRO- Au(24)Co(8) model presents a pseudo-gap in the minority-spin channel of the Au *sp* PDOS, such a feature allows attributing the absorption peak at ≈ 2.6 eV in the imaginary part of the dielectric function (Supplementary Figure 12) to a *d*-*sp* interband transition enhanced by the localization of the *sp* band.

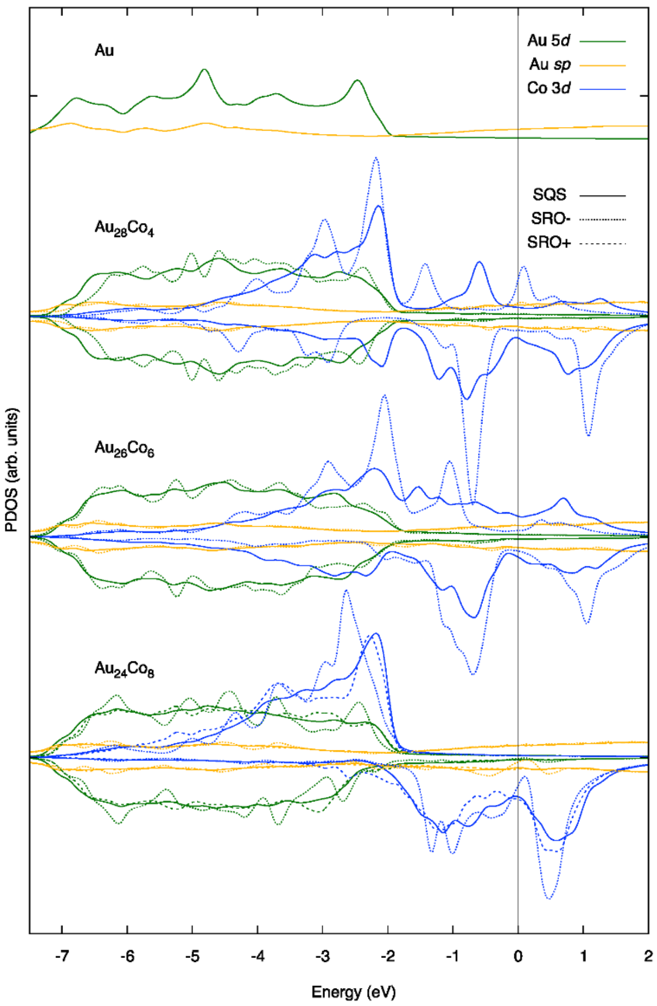

Supplementary Figure 11. **Projected DOS.** Projected DOS computed at the LDA+U level of theory. PDOS were aligned to the Fermi level. Colors in the graphs are: green (Au 5*d*), orange (Au *sp*), blue (Co 3*d*), black

continuous line (SQS), black dotted line (SRO-), black dashed line (SRO+). Source data are provided as a Source Data file.

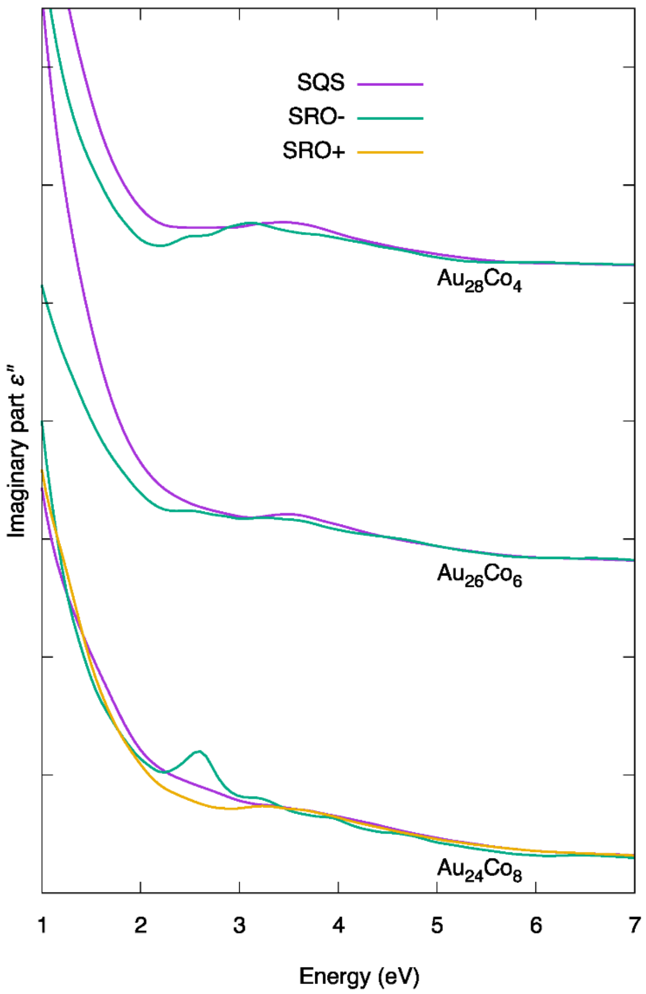

Supplementary Figure 12. Imaginary part of the dielectric function. Imaginary part of the dielectric functions calculated for SRO- (green), SRO+ (light brown) and SQS (purple) cell models. Source data are provided as a Source Data file.

Suppl. Note 8
Additional characterization data of NPs samples.

Sample	Composition	Size (nm)
A	Au(76)Co(24)	45 ± 18
B	Au(79)Co(21)	24 ± 6
C	Au(87)Co(13)	6 ± 3
D	Au(80)Fe(20)	39 ± 12
E	Au(83)Fe(17)	23 ± 6
F	Au(45)Ag(55)	35 ± 13
G	Au(45)Ag(55)	22 ± 6
Au49	Au	49 ± 18
Au29	Au	29 ± 8
Au9	Au	9 ± 4

Supplementary Table 5. List of all samples of this study. The samples name, composition and size are listed below.

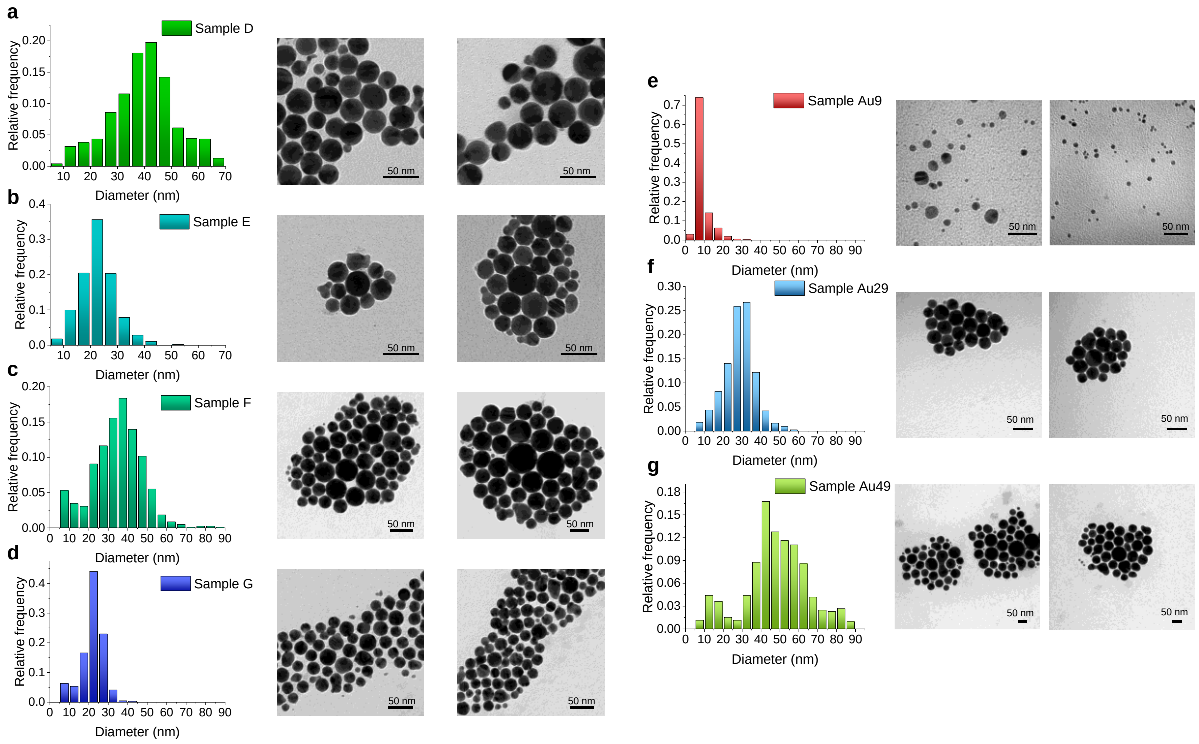

Supplementary Figure 13. TEM characterization of NPs. Size histograms and TEM images of the Au-Fe (**a-b**), Au-Ag (**c-d**) and Au (**e-f-g**) NPs samples of this study. Statistics considered >500 NPs for each sample. Source data are provided as a Source Data file.

Supplementary references

1. Olmon, R. L. *et al.* Optical dielectric function of gold. *Phys. Rev. B* **86**, 235147 (2012).
2. McPeak, K. M. *et al.* Plasmonic films can easily be better: Rules and recipes. *ACS Photonics* **2**, 326–333 (2015).
3. Johnson, P. & Christy, R. Optical constants of transition metals: Ti, V, Cr, Mn, Fe, Co, Ni, and Pd. *Phys. Rev. B* **9**, 5056–5070 (1974).
4. Avakyan, L. *et al.* Theoretical approach for calculation of dielectric functions of plasmonic nanoparticles of noble metals, magnesium and their alloys. *Opt. Mater.* **109**, 110264 (2020).
5. Kuisma, M., Ojanen, J., Enkovaara, J. & Rantala, T. T. Kohn-Sham potential with discontinuity for band gap materials. *Phys. Rev. B* **82**, 115106 (2010).
6. Rahm, J. M. *et al.* A Library of Late Transition Metal Alloy Dielectric Functions for Nanophotonic Applications. *Adv. Funct. Mater.* **30**, (2020).
7. Bubaš, M. & Sancho-Parramon, J. DFT-Based Approach Enables Deliberate Tuning of Alloy Nanostructure Plasmonic Properties. *J. Phys. Chem. C* **125**, (2021).
8. Marzari, N., Ferretti, A. & Wolverton, C. Electronic-structure methods for materials design. *Nat. Mater. 2021 206* **20**, 736–749 (2021).
9. Lee, Y. P., Kudryavtsev, Y. V, Nemoshkalenko, V. V, Gontarz, R. & Rhee, J. Y. Magneto-optical and optical properties of Fe-rich Au-Fe alloy films near the fcc-bcc structural transformation region. *Phys. Rev. B* **67**, 104424 (2003).
10. Gong, C. & Leite, M. S. Noble Metal Alloys for Plasmonics. *ACS Photonics* **3**, 507–513 (2016).
